# Matrine attenuates endoplasmic reticulum stress and mitochondrion dysfunction in nonalcoholic fatty liver disease by regulating SERCA pathway

**DOI:** 10.1186/s12967-018-1685-2

**Published:** 2018-11-20

**Authors:** Xiaobo Gao, Shun Guo, Song Zhang, An Liu, Lei Shi, Yan Zhang

**Affiliations:** Department of Pharmacy, The Second Affiliated Hospital of Air Force Medical University, 569 N. Xinsi Road, Xi’an, 710038 China

**Keywords:** Matrine, ER stress, Ca^2+^ homeostasis, Mitochondrion dysfunction, Nonalcoholic fatty liver disease

## Abstract

**Background:**

Endoplasmic reticulum (ER) stress, which can promote lipid metabolism disorders and steatohepatitis, contributes significantly to the pathogenesis of nonalcoholic fatty liver disease (NAFLD). Calcium (Ca^2+^) homeostasis is considered to play a key role in ER stress. Matrine (Mat) has been applied for the treatment of hepatitis B, but its effect on NAFLD is still unknown, and there is no unified view of Mat on the regulation of ER stress in the previous literature.

**Methods:**

The pharmacological effects were studied in high-fat-diet or methionine–choline-deficient diet induced C57BL/6J mice models and in palmitic acid (PA) induced L02 human liver cell model. Calcium fluorescence experiments, computational virtual docking analysis and biochemical assays were used in identifying the locus of Mat.

**Results:**

The results showed that Mat-treated mice were more resistant to steatosis in the liver than vehicle-treated mice and that Mat significantly reduced hepatic inflammation, lipid peroxides. The beneficial effect of Mat was associated with suppressing ER stress and restoring mitochondrial dysfunction. Additionally, Mat decreased the PA-induced lipid accumulation, ER stress and cytosolic calcium level ([Ca^2+^]_c_) in hepatocyte cell lines in low and middle dose. However, the high dose Mat did not show satisfactory results in cell model. Calcium fluorescence experiments showed that Mat was able to regulate [Ca^2+^]_c_. By computational virtual docking analysis and biochemical assays, Mat was shown to influence [Ca^2+^]_c_ via direct inhibition of SERCA.

**Conclusions:**

The results showed that the bi-directional regulation of Mat to endoplasmic reticulum at different doses was based on the inhibition of SERCA. In addition, the results also provide a theoretical basis for Mat as a potential therapeutic strategy in NAFLD/NASH.

**Electronic supplementary material:**

The online version of this article (10.1186/s12967-018-1685-2) contains supplementary material, which is available to authorized users.

## Background

Nonalcoholic fatty liver disease (NAFLD) is a clinicopathological condition, which is defined as excessive fat accumulation and formation of lipid droplets in the cytoplasm of hepatocytes, accompanied by enlargement of liver and inflammation. It ranges from simple steatosis to nonalcoholic steatohepatitis (NASH) and could eventually lead to cirrhosis and hepato-cellular carcinoma [1, 2]. Currently, it has become clear that NALFD is a multifactorial disease closely related to liver steatosis, insulin resistance, oxidative stress, inflammatory reaction, etc. Persistent endoplasmic reticulum (ER) stress and mitochondrial dysfunction participate in the regulation of the above physiological changes and both play an important role in the progression from NALFD to NASH [3, 4].

Calcium ion (Ca^2+^), a critical and versatile intracellular secondary messenger, is involved in various cellular processes. ER is known to be the most important intracellular Ca^2+^ store [5]. The abnormal release of ER Ca^2+^ not only induces ER stress and mitochondrial dysfunction, but also exacerbates the hepatic cell lipotoxicity [6]. Sarcoplasmic/endoplasmic reticulum calcium ATPase (SERCA) pump, the main regulator of intracellular Ca^2+^, actively re-accumulates released Ca^2+^ back into the ER, and therefore maintains Ca^2+^ homeostasis. SERCA activity is reduced in NALFD, while enhanced SERCA activity alleviates ER stress and apoptosis [7, 8]. Recent studies have showed that the homeostasis of Ca^2+^ is closely related to the development of NALFD to NASH [9–11].

Natural monomers or extracts isolated from plants or herbs have been demonstrated to effectively treat various diseases with relatively low toxicity. Matrine (Mat), a tetracyclo-quinolizidine alkaloid, is mainly derived from leguminosae such as *Sophora flavescens* and *Sophora subprostrata*. Recent researches have shown that Mat can exert a wide range of pharmacological effects and has the potential to treat a variety of diseases, including cardiac fibrosis, parkinson’s disease and arthritis [12–14]. In addition, the clinical drugs based on Mat have been applied for the treatment of hepatitis, and it has been reported that the intramuscular injection of Mat effectively improves the clinical symptoms of patients with chronic hepatitis B, recovers liver function and alters serum conversion from positive to negative hepatitis B virus DNA [15]. In addition, some studies have shown that Mat and oxymatrine (oxy-Mat) can inhibit the endoplasmic reticulum stress in steatohepatitis and sodium arsenite induced hepatic injury, but other reported that Mat can induce the ER stress and promote tumor cell apoptosis [16, 17]. Then what is the role of Mat in non-alcoholic fatty liver disease and in the relationship between ER stress response and calcium homeostasis? The present study investigated accordingly whether Mat has a therapeutic effect on NALFD, and made a preliminary inquiry into the molecular mechanisms involved.

## Methods

### Materials

Matrine (M5319), palmitic acid (V900121), 2-Aminoethoxydiphenyl borate (D9754) and thapsigargin (T9033) were purchased from Sigma-Aldrich (St. Louis, MO, USA). The antibodies included anti-Phospho-PERK (Thr980) (#3179), anti-PERK (#3192) anti-CHOP (#2895), anti-BiP (#3177) and anti-cleaved caspase3 (#9664) were purchased from Cell Signaling Technology (USA). GAPDH (AT0002, CMCTAG, USA). Other antibodies included anti-ATF6 (ab37149), anti-phospho-IRE1 (phospho S724) (ab48187), anti-IRE1 (ab37073), anti-SREBP1 (ab28481), Anti-Fatty Acid Synthase (ab22759), anti-Acetyl Coenzyme A Carboxylase (ab45174), anti-NF-κB p65, (ab16502), anti-c-jun (phospho S63) (ab32385) were purchased from Abcam (Cambridge, UK).

### Animals and experimental designs

The vivo studies were performed in male C57BL/6J (3–4 weeks) mice, which were purchased from the animal center of the Air Force Medical University (Xi’an, China). The mice were kept under a light-controlled condition (12 h/12 h light/dark cycle) in a special room with constant temperature (23 ± 2 °C) and humidity (55 ± 15%), and were free to eat and drink. For the HFD-fed mice, mice were acclimated for 2 weeks and randomly divided into 5 groups (n = 10). The control group received regular chow, whereas the HFD group received the high-fat diet (TP-26301, Trophic, Nantong, China) for 12 weeks, and HFD + Mat groups (HFD + Mat L, M, H) received high-fat diet for 4 weeks and then combined with Mat (0.5 mg/kg, 2.5 mg/kg, 10 mg/kg, respectively) intervention from 5- to 12-week. For the MCD diet-fed mice, mice were acclimated for 2 weeks and divided into 5 groups (n = 10). The control group received regular chow, whereas the MCD group received the MCD diet (TP-3005G, Trophic, Nantong, China) for 6 weeks, and MCD + Mat groups (MCD + Mat L, M, H) received MCD diet for 2 weeks and then combined with Mat (0.5 mg/kg, 2.5 mg/kg, 10 mg/kg, respectively) intervention from 3- to 6-week. In the end of experiment, all the mice were euthanized after a 12-h fast, and the liver tissues and blood samples were collected for evaluation.

### Biochemical analysis and histopathology

Serum triglyceride (TG), total cholesterol (TC), alanine aminotransferase (ALT), and aspartate aminotransferase (AST) were assessed by automatic biochemical analyzer (200FR, TOSIBA, Japan). Pro-inflammatory cytokines (TNF-α, IL-6, IL-10) in serum were assessed with commercial kits based on colorimetric method. Liver tissue in 4% paraformaldehyde was stained with hematoxylin and eosin (H&E) to observe the damage and was subjected to oil red O staining to visualize lipid droplets in the liver. The images were captured and analyzed by a fluorescence microscope (NI, Nikon, Tokyo, Japan).

### Cell culture

L02 human liver cells (a kind gift from the professor Zhang of Air Force Medical University) were used for in vitro study. L02 cells were cultured in Dulbecco’s Modified Eagle medium (DMEM) supplemented with 10% (v/v) fetal bovine serum (FBS) and 1% penicillin/streptomycin at 37 °C in a humidified atmosphere of 5% CO_2_. Future experiments were conducted after the completion of cell processing.

### Oil red O staining

The cultured L02 cells grown on cell plates were washed with phosphate-buffered saline (PBS) three times and fixed with 4% paraformaldehyde for 1 h. Then, the fixed cells were washed with 60% isopropanol for 30 s and PBS three times. In darkness, the cells were stained with freshly diluted Oil Red O working solution for 1 h at 37 °C, after which they were counterstained with hematoxylin for 1 min. The cells were washed with PBS three times and ultimately observed and photographed by an inverted fluorescence microscope (IX53, Olympus, Tokyo, Japan).

### ROS detection

Intracellular ROS levels were detected as the manufacturer’s instructions described. L02 cells were cultured on cell plates with suitable density. After the corresponding treatment, cells were washed with serum-free medium for three times, and incubated with 10 μM 21,71-dichlorofluorescin diacetate (S0033, Beyotime Biotechnology, Jiangsu, China) in serum-free medium at 37 °C for 30 min. Then, cells were washed with the same serum-free medium for three times. Imaging was observed and photographed by the fluorescence microscope. DCFH fluorescence was measured by fluorescence spectrophotometer (excitation at 488 nm and emission at 525 nm, 180 manual).

### Immunohistochemistry

ER histochemistry was detected using Streptavidin Rabbit HRP Kit (CW2035, CWBIO, Beijing, China), according to the manufacturer’s instructions. HRP can catalyze the color development of substrates, thus inferring the presence and distribution of antigens to be detected. Primary antibodies were CHOP, GRP78 and ATF6. Secondary antibodies for fluorescent imaging were tagged with the corresponding species to the primary antibody. Imaging was observed and photographed by the fluorescence microscope.

### Apoptosis assay

Apoptosis was detected using Annexin V-FITC/PI cell apoptosis detection kit (C1063, Beyotime Biotechnology, Jiangsu, China). Cells from different groups were digested with trypsin without EDTA, resuspended in the binding buffer, stained with Annexin V-FITC for 15 min and PI for 5 min, and then analyzed by flow cytometry (Novocyte 2040R, ACEA, USA).

### Measurement of cytosolic Ca^2+^

Cytosolic Ca^2+^ ([Ca^2+^]_c_) in L02 cells was measured using the Fura-3AM fluorescent indicator (S1056, Beyotime Biotechnology, Jiangsu, China) as described in the specifications. Briefly, L02 cells seeded in 96-well plates or 6-well plates and received the corresponding treatment respectively. For measuring the [Ca^2+^]_c_, cells were washed and incubated with 5 μM Fura-3/AM and 0.1% Pluronic F-127 (ST501, Beyotime) in Hanks’ balanced salt solution (HBSS) for 45 min at 37 °C. The cells were then washed with PBS three times. Fluorescence was subsequently measured by fluorescence spectrophotometer (Tecan infinite M200 Pro, Switzerland) (excitation at 488 nm and emission at 530 nm, 180 manual). Background fluorescence was subtracted from all signals. Changes in [Ca^2+^]_c_ are represented by changes in fluorescence expressed as: F/F_0_, where F_0_ is control fluorescence.

### Measurement of mitochondrial membrane potential

Mitochondrial membrane potential was detected using mitochondrial membrane potential assay kit with JC-1 (C2006, Beyotime Biotechnology, Jiangsu, China), according to the manufacturer’s instructions. JC-1 staining was observed and photographed by the fluorescence microscope. In addition, its fluorescence was measured by fluorescence spectrophotometer.

### Western blotting

Total protein was separated by SDS-PAGE (Bio-Rad, CA, USA) and electro-transferred onto the polyvinylidene difluoride (PVDF) membranes. Further, the PVDF membranes were incubated with specific primary antibodies (1:1000) overnight at 4 °C. The appropriate secondary antibodies (1:4000) were used to tag primary antibody for 3 h and an enhanced chemiluminescence (ECL) detection system (Millpore immobilonTM HRP Substrate) was used to develop the immunoreactive bands. Finally, the protein band densities were quantified with Fusion software.

### Molecular docking studies

The docking studies were carried out using the AutoDock Vina software. The input files were prepared in the graphic interface AutoDock Tools 1.5.2 [18]. The protein crystal structure of rabbit SERCA1a (PDB code: 4BEW) and SERCA2 (PDB code: 5MPM) was downloaded from the protein data bank as a basis for modeling. All the ligands in the computational study were converted into 3-D format. The docking parameters were as follows: blind docking procedure with a grid box of 126 × 126 × 126 Å^3^ and a grid spacing of 0.375 Å. For the scoring we selected the Lamarckian Genetic Algorithm with maximum number of energy evaluations of 1 × 10^7.^

### SERCA activity analysis

SERCA activity was analyzed using the Ca^2+^-ATP enzyme assay kit (Biological Technology, Beijing, China) according to the manufacturer’s instructions. Protein concentration was determined by the BCA protein assay kit, and the amount of inorganic phosphate reflects SERCA activity.

### Statistical analysis

Dates in this study were expressed as the mean ± standard error of mean (SEM). Statistical analyses were performed with independent t-test, one-way ANOVA, followed by a least significant difference post hoc analysis. For all analysis, *P* values < 0.05 were considered statistically significant.

## Results

### Effect of Mat on lipid metabolic derangements in HFD mice

**Fig. 1:**
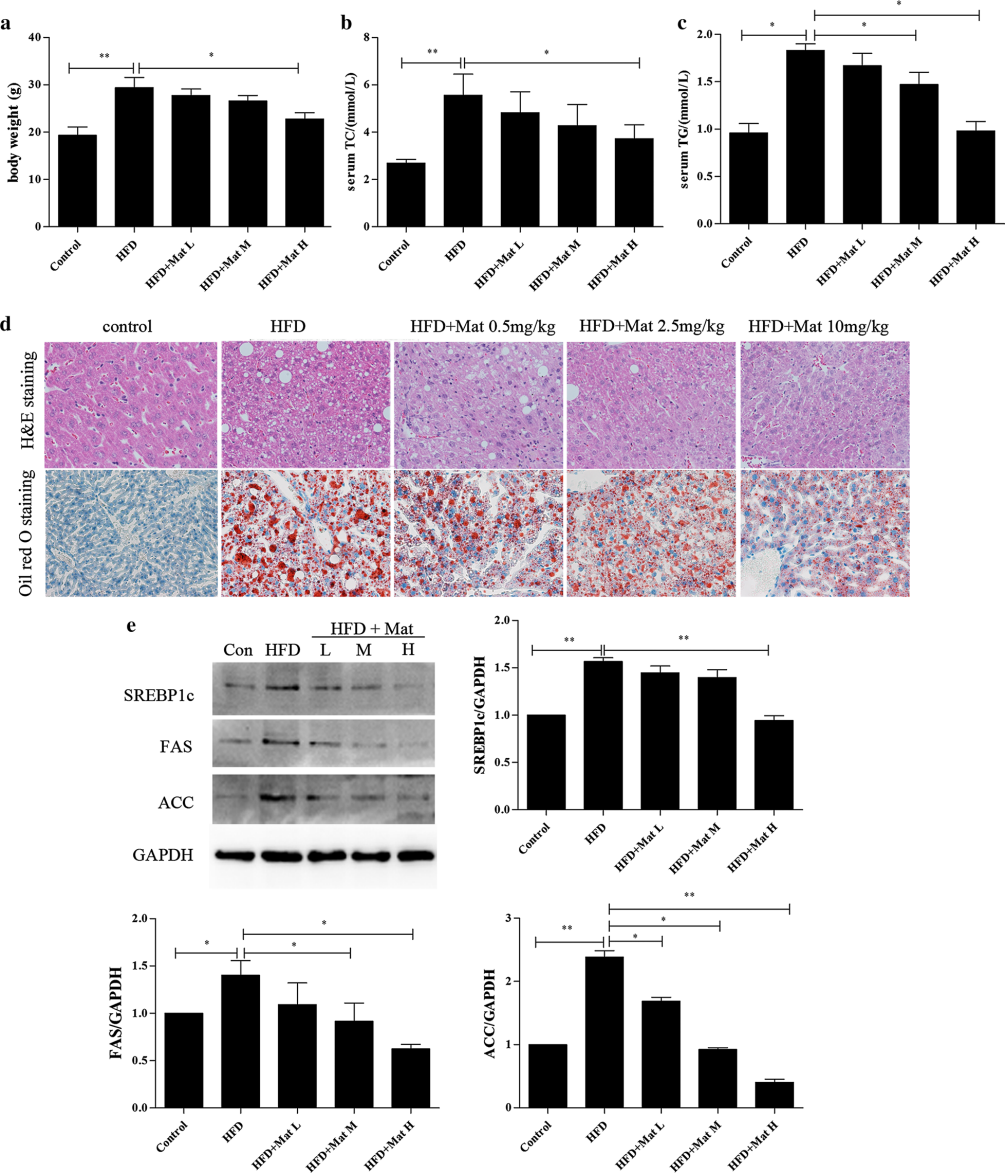
Matrine (Mat) ameliorated body weight and lipid metabolic derangements in HFD mice (n = 10 animals per condition). **a** Body weight, **b** serum total cholesterol (TC) levels. **c** Serum triglyceride (TG) levels. **d** HE staining (×200) and Oil red O staining (×200) of livers in HFD mice. **e** Expressions of lipogenesis proteins (SREBP1c, FAS and ACC) in livers. HFD + Mat groups (L, M, H) represent high-fat diet combined with Mat 0.5 mg/kg, 2.5 mg/kg and 10 mg/kg, respectively.*P < 0.05 and **P < 0.01

The high-fat diet has been extensively used to produce a diet-induced model of NAFLD in animals [19, 20]. After 12 weeks feeding, body weight of the HFD mice was significantly increased compared to the normal diet group. Mat administration successfully reduced the HFD-induced body weight (Fig. 1a). Compared with control group, serum TC and TG levels were notably increased in HFD mice, while Mat treatment could significantly reduce this effect (Fig. 1b, c). Using HE staining and oil red O staining, the liver sections showed apparent inflammatory damage and lipid droplets within the lobule in HFD mice, both of which were attenuated in Mat-treated HFD mice (Fig. 1d). Steatosis is considered an important feature of NASH and has been identified as a risk factor for liver fibrosis [21]. Our results showed that Mat down-regulated the proteins levels of sterol regulatory element binding protein 1c (SREBP-1c), fatty acid synthase (FAS), and acetyl-coenzyme A-carboxylase (ACC) in HFD mice, all of which are closely related to lipid production (Fig. 1e). These results indicated that Mat could attenuate hyperlipidemia, hepatic steatosis and hepatic lipogenesis in NAFLD.

### Effect of Mat on ER stress and inflammatory responses in MCD-diet mice

**Fig. 2:**
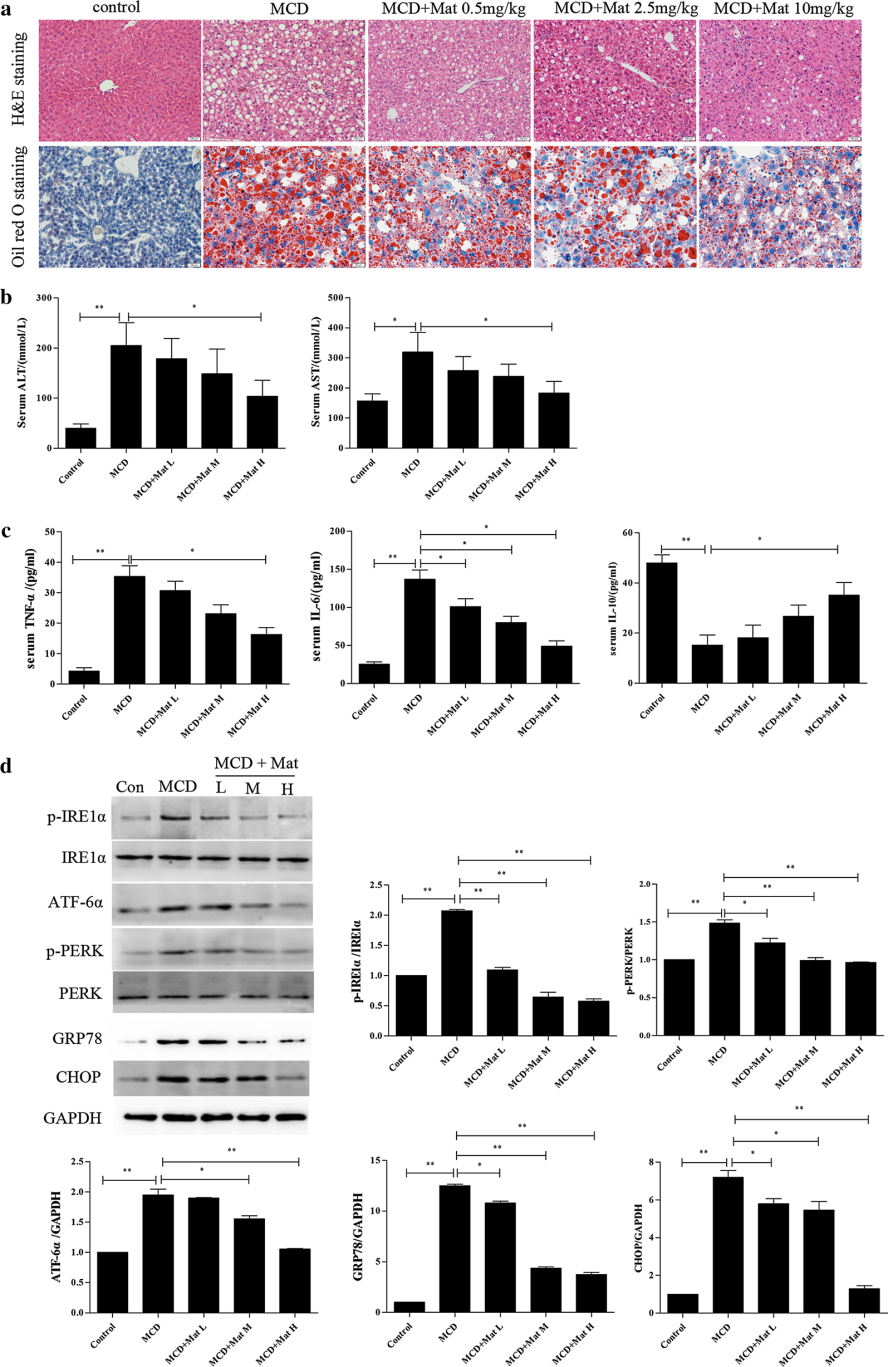
Mat alleviates ER stress and inflammatory responses in MCD mice (n = 10 animals per condition). **a** HE staining (×200) and Oil red O staining (×200) of livers in MCD mice. **b** Serum ALT and AST levels (markers of hepatic injury). **c** Serum TNF-α, IL-6 and IL-10 (inflammatory factors) levels. **d** Western blot analysis for expression of ER stress signal proteins in liver: p-PERK, p-IRE1, PERK, IRE1, ATF6, GRP78 and CHOP. *P < 0.05 and **P < 0.01

Compared with HFD group, mice in MCD group showed more severe liver injury. Much larger vacuoles were found in MCD mice with H&E staining, Mat treatment significantly relieved the pathological changes (Fig. 2a). Moreover, Mat treatment made larger lipid droplets turn to small and reduced the amount of lipid droplets in MCD mice (Fig. 2a). Consistent with the histological results, the serum ALT and AST levels, two markers of hepatic injury, were obviously decreased by Mat in MCD mice (Fig. 2b).

Inflammatory response is an important part of the pathogenesis of NASH. Compared to the control group, the levels of serum TNF-α, and IL-6 were significantly increased in MCD mice, while the IL-10 levels were decreased. In contrast, Mat treatment successfully restored the expression of these inflammatory cytokines in MCD mice (Fig. 2c).

ER stress plays a catalytic role in the development of NASH [22, 23]. We examined the expression levels of major ER stress-related proteins in order to inspect whether ER stress signals are involved in the Mat-ameliorating mechanism on NASH. Compared to the control group, the ER stress related protein levels of p-IRE1α, ATF6α, GRP78, p-PERK and CHOP were significantly increased in the liver of MCD mice, while Mat administration could inhibit this effect (Fig. 2d).

### Effect of Mat on lipid accumulation in palmitate acid (PA)-induced L02 cells

**Fig. 3:**
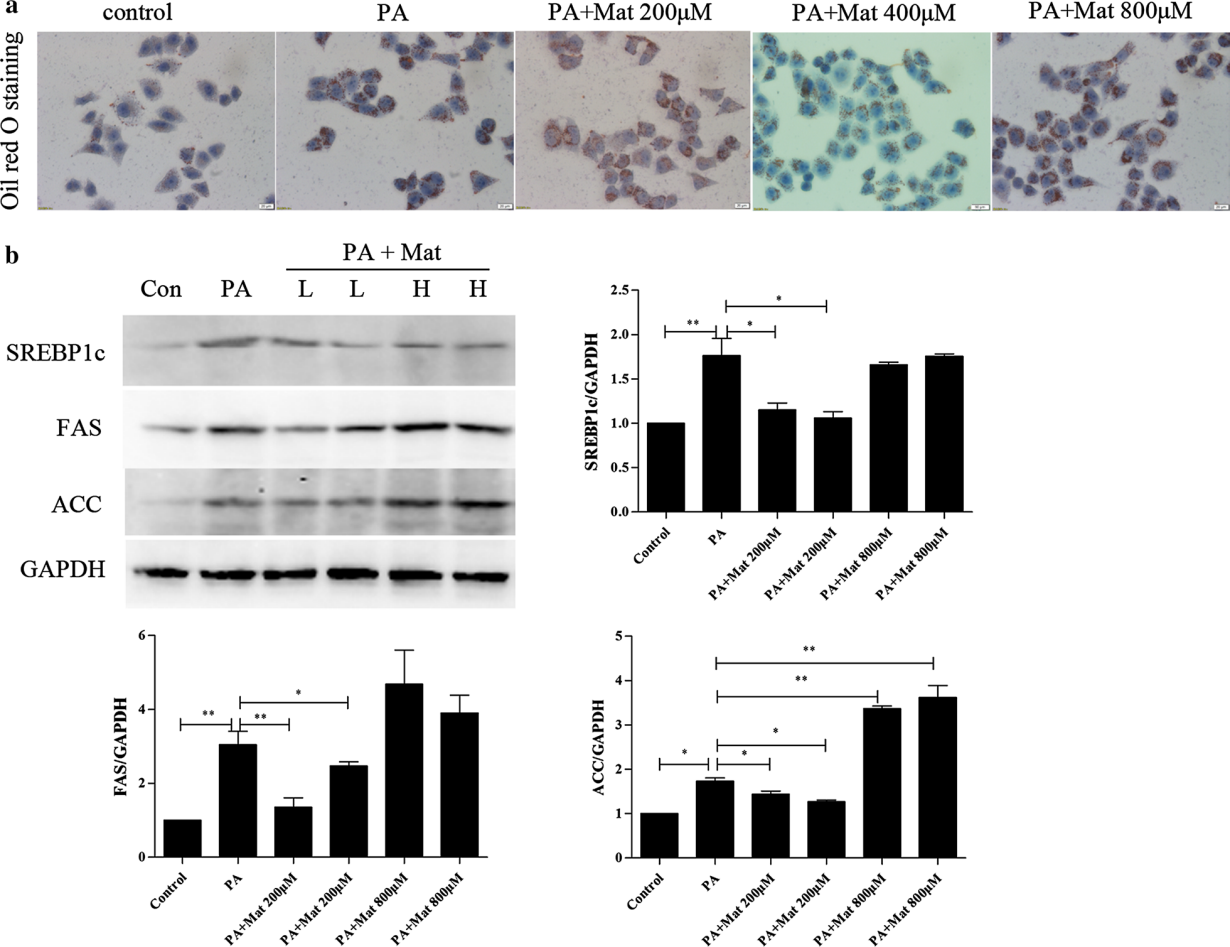
Effect of Mat on lipid metabolism in palmitate acid (PA)-induced L02 cells. The L02 cells were treated with PA (500 μM) or the combination of PA (500 μM) and Mat (200, 400, 800 μM) for 12 h. **a** Oil red O staining (×400) of L02 cells. **b** Western blot analysis for expression of SREBP1c, FAS, and ACC in L02 cells (lipogenesis proteins). PA + Mat groups (L, H) represent PA (500 μM) combined with Mat 200 μM and 800 μM, respectively. *P < 0.05 and **P < 0.01

Palmitate exhibited significant lipotoxicity in a dose-dependent manner in human hepatocytes [24]. Using oil red O staining, profound lipid droplets were found in PA-induced L02 cells. Different from the high dose Mat (800 μM) group, low and middle dose Mat (200 and 400 μM) treatment effectively decreased lipid droplets in PA-induced L02 cells (Fig. 3a). Intracellular TG, TC accumulations were also detected by biochemical assays. The results also showed that low and middle dose Mat (200 and 400 μM) can better decreased lipid droplets, but nor high dose (Additional file 1: Figure S2). Moreover, the protein levels of SREBP-1c, FAS, and ACC in PA-induced L02 cells were significantly down-regulated by low dose Mat, which was consistent with the results of mice (Fig. 3b). These results indicated that Mat could inhibit lipid accumulation in PA-induced L02 cells.

### Effect of Mat on ER stress in PA-induced L02cells

**Fig. 4:**
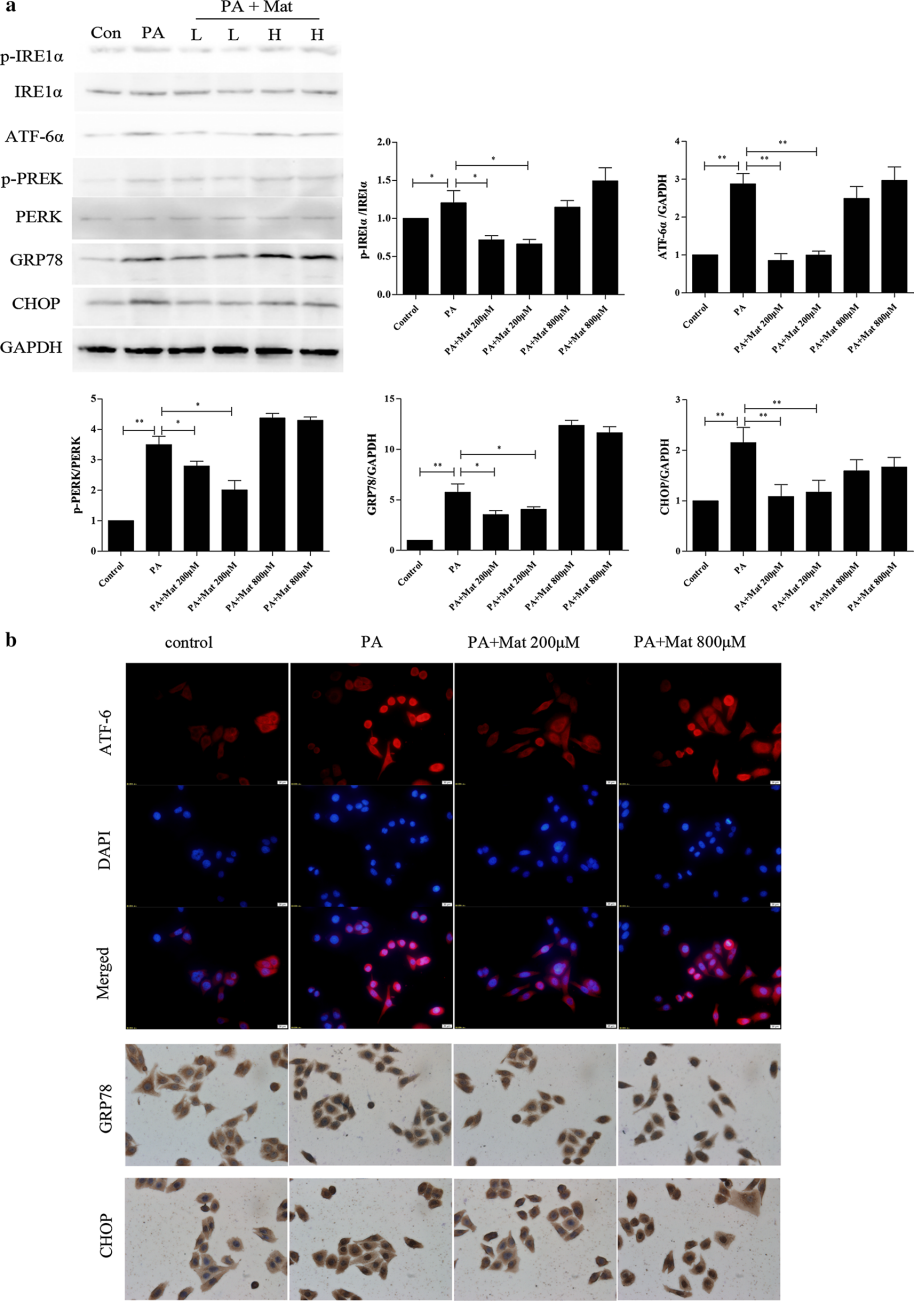
Effect of Mat on ER stress in PA-induced L02 cells. The L02 cells were treated with PA (500 μM) or the combination of PA (500 μM) and Mat (200, 400, 800 μM) for 12 h. **a** Western blot analysis for expression of ER stress signal proteins in L02 cells:p-PERK, p-IRE1, PERK, IRE1, ATF6, GRP78 and CHOP. PA + Mat groups (L, H) represent PA (500 μM) combined with Mat 200 μM and 800 μM, respectively. **b** ER histochemistry (×400) of ATF6, GRP78 and CHOP. *P < 0.05 and **P < 0.01

PA treatment can bring about significant ER stress in L02 cells [25]. Compared to the control group, the protein levels of p-IRE1α, ATF6α, GRP78, p-PERK and CHOP were up-regulated in PA-induced L02 cells (Fig. 4a). This effect was decreased by low dose Mat, but not by the high dose Mat. Furthermore, the ER histochemistry results showed that Mat significantly inhibited the GRP78, CHOP expression and the entry of ATF-6 into the nucleus compared with the model group (Fig. 4b). These findings demonstrated that Mat treatment could block the ER stress-induced subsequent reactions, which might be a mechanism mediating Mat’s action in alleviating NAFLD/NASH.

### Effect of Mat on mitochondrial damage, ROS production and apoptosis in PA-induced L02 cells

**Fig. 5:**
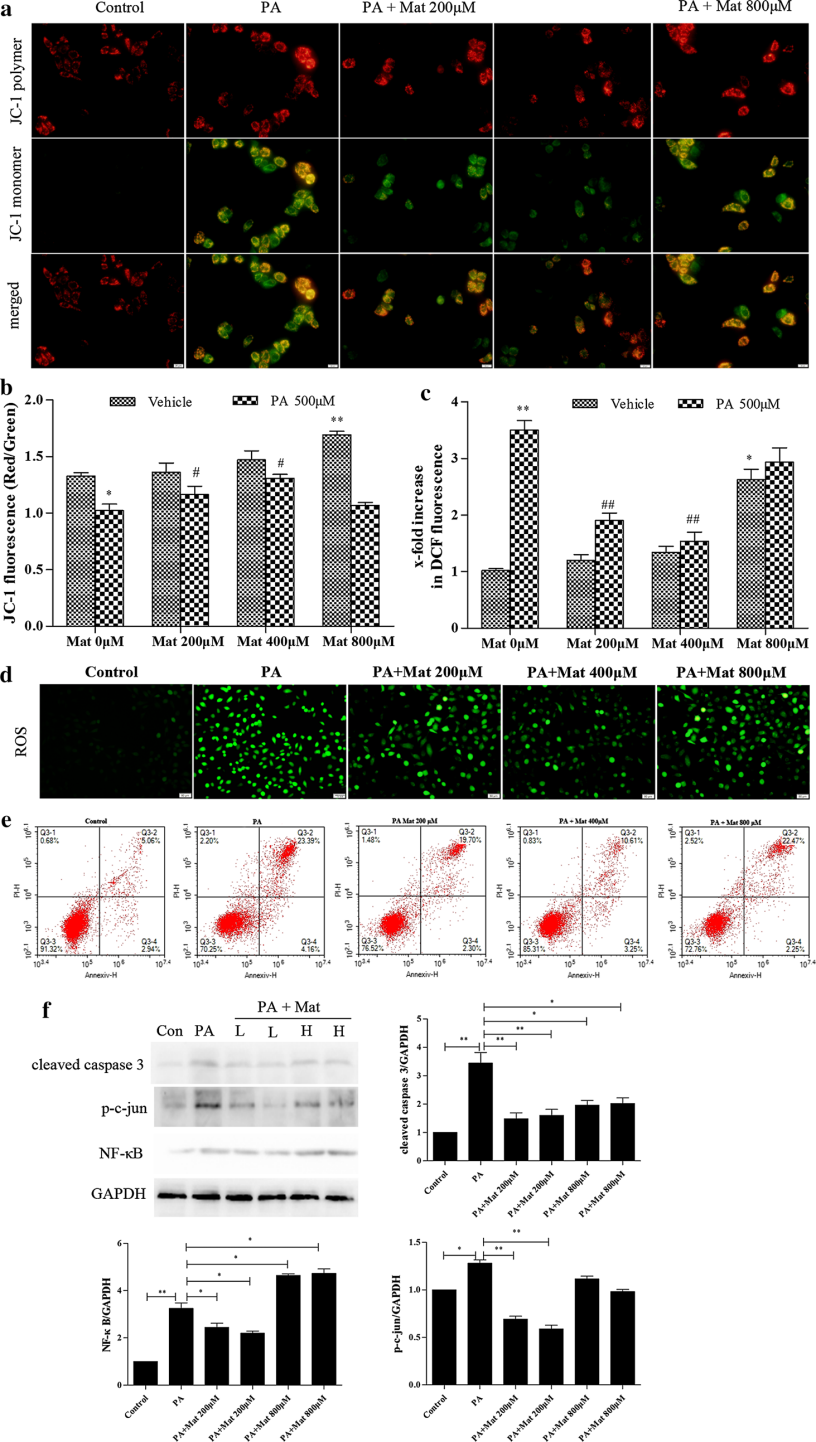
Effect of Mat on mitochondrial activation, ROS production and apoptosis in PA-induced L02 cells. The L02 cells were treated with PA (500 μM), Mat (200, 400, 800 μM) or the combination of PA (500 μM) and Mat (200, 400, 800 μM) for 12 h. **a** Mitochondrial membrane potential (MMP) imaging (×400). **b** JC-1 fluorescence and **c** DCF fluorescence detected by fluorescence spectrophotometer. *P < 0.05 and **P < 0.01 vs. Control, ^#^P < 0.05 and ^##^P < 0.01 vs. PA. **d** ROS imaging (×400). **e** apoptosis analyzed by flow cytometry. **f** expression of cleaved caspase 3, p–c-jun and NF-κB in L02 cells. *P < 0.05 and **P < 0.01

The decline of mitochondrial membrane potential (MMP) is a landmark event in the early stage of apoptosis. The activation of mitochondrial damage pathway plays a key role in PA mediated lipid apoptosis [26]. Obviously, MMP was decreased in PA-induced cells (Fig. 5a, b). However, low and middle dose Mat significantly increased the MMP in model cells, suggesting that PA-mediated mitochondrial activation was inhibited by low and middle dose Mat, not the high dose Mat.

ER stress and mitochondrial dysfunction could lead to ROS production in the cells, and then the abundant production of ROS exacerbated mitochondrial damage and apoptosis [27]. A large amount of ROS was produced in L02 cells by PA treatment and ROS levels in PA-induced L02 cells were reduced in the presence of Mat (Fig. 5d). In addition, as shown in Fig. 5c, the low dose Mat had the obvious effect second to the middle dose Mat, while the high dose Mat had a minor effect.

ER stress is known to promote apoptosis [28]. We investigated the effects of Mat on apoptosis by a flow cytometry. The results showed that Mat reduced the apoptosis in PA-induced L02 cells and the reduction effect of middle dose Mat was the strongest among three concentrations, while the high dose Mat only had limited effect (Fig. 5e). We also detected the expression of apoptosis related proteins cleaved caspase 3, p–c-jun and NF-κB in PA-induced L02 cells and the result was consistent with that in flow cytometry (Fig. 5f).

### Effect of Mat on cytosolic Ca^2+^ level ([Ca^2+^]_c_) in L02 cells

**Fig. 6:**
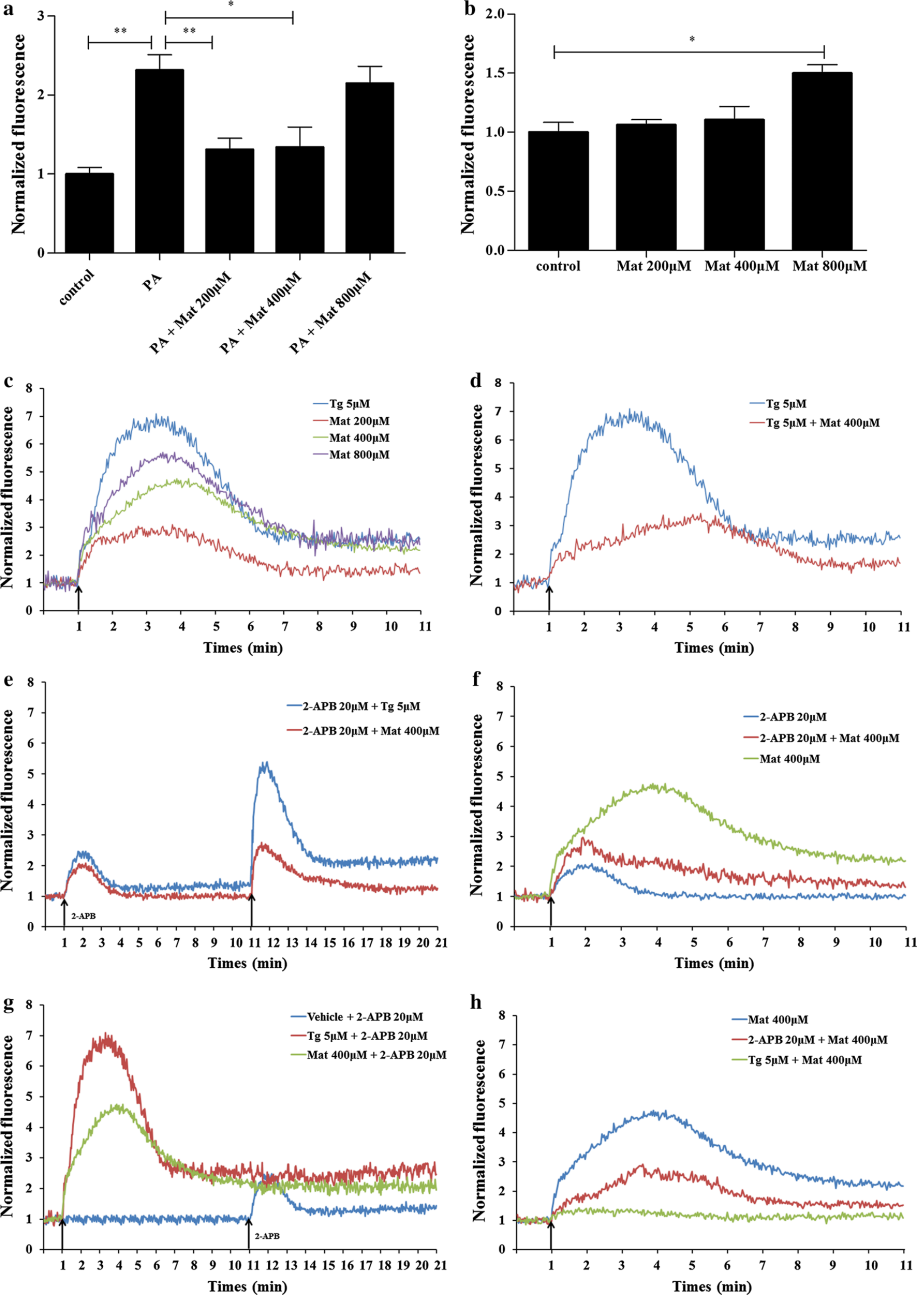
Effect of Mat on cytosolic Ca^2+^ level in L02 cells. In the prolonged experiments (**a** and **b**), the L02 cells were treated with PA (500 μM), Mat (200, 400, 800 μM) or the combination of PA (500 μM) and Mat (200, 400, 800 μM) for 12 h. Then the cells were incubated with the Fura-3AM fluorescent indicator and measured by fluorescence spectrophotometer. **a** the fluorescence of the combination of PA and Mat. **b** the fluorescence of Mat incubated alone. *P < 0.05 and **P < 0.01. In the instantaneous stimulation experiments (**c**–**h**), the L02 cells were incubated with the Fura-3AM fluorescent indicator and then treated by 2-APB (20 μM), Tg (5 μM) and Mat (200, 400, 800 μM). **c** Mat-induced instant Ca^2+^ release of L02 cells. The Ca^2+^ curves represent an average of three independent experiments. **d** the combined addition of Mat and Tg (SERCA inhibitor). **e** reduction of Mat or Tg-induced Ca^2+^ release by pretreatment with 2-APB (IP_3_R inhibitor) for 10 min. **f** the together addition of Mat and 2-APB. **g** the Ca^2+^ curves induced by 2-APB after vehicle, Tg or Mat pretreatment for 10 min. **h** reduction of Mat-induced Ca^2+^ release by pretreatment with Tg or 2-APB for 12 h

We examined the effect of Mat treatment on [Ca^2+^]_c_ in L02 cells. After the 12 h prolonged incubation of PA, the [Ca^2+^]_c_ was found to be increased in L02 cells compared to the control group. Low and middle dose Mat treatment could reduce the [Ca^2+^]_c_ increased by PA, but high dose Mat had little effect (Fig. 6a). Interestingly, when Mat was incubated alone in normal L02 cells, low dose did not increase the level of [Ca^2+^]_c_. On the contrary, High dose Mat increased [Ca^2+^]_c_ in L02 cells (Fig. 6b).

In order to further explain the mechanism of Mat, Tg (SERCA inhibitor) and 2-APB (IP_3_R inhibitor) were used to detect the effect of Mat on the level of cytosolic Ca^2+^ ([Ca^2+^]_c_) in instantaneous stimulation experiments. In these trials, normal L02 cells were imaged for 1 min before the first treatment was added in order to establish a baseline. Subsequently, the cells were stimulated with different reagents. We observed that the addition of Mat to L02 cells in the absence of extracellular Ca^2+^ provoked a rapid, concentration-dependent increase in [Ca^2+^]_c_ reaching a Ca^2+^ peak and then descending to a sustained plateau above basal levels (Fig. 6c). Tg, the potent inhibitor of SERCA pump, elicited a similar Ca^2+^ response but with a higher Ca^2+^ peak at 5 μM. However, the combined addition of Mat and Tg led to a lower Ca^2+^ release in L02 cells compared to the addition of Tg (Fig. 6d).

To confirm that ER Ca^2+^ pool is the source of the increase in [Ca^2+^]_c_ caused by Mat, we observed the changes of [Ca^2+^]_c_ induced by 2-APB under different conditions. Both 2-APB pretreatment for 10 min and the combined addition are able to reduce the Ca^2+^ peak provoked by Tg or Mat (Fig. 6e, f). 2-APB can induce the release of ER Ca^2+^ only when ER Ca^2+^ store is replete [29]. A slight Ca^2+^ wave was observed when 20 μM 2-APB stimulated L02 cells, while the wave was disappeared when cells were stimulated by Tg pretreatment for 10 min, as well as by Mat pretreatment for 10 min (Fig. 6g). Moreover, to confirm that SERCA is the source of the increase in [Ca^2+^]_c_ caused by Mat, we pretreated the cells with 5 μM Tg for 12 h. Tg pretreatment significantly prevented the increase of [Ca^2+^]_c_ induced by Mat. Nevertheless, 2-APB pretreatment only partly reduced the increase of [Ca^2+^]_c_ induced by Mat (Fig. 6h), suggesting that Mat induced [Ca^2+^]_c_ increase mainly through the regulation of SERCA channels. Altogether, these data demonstrate that Mat is able to modulate the calcium homeostasis of L02 cells.

### Mat regulates Ca^2+^ via the SERCA pathway

**Fig. 7:**
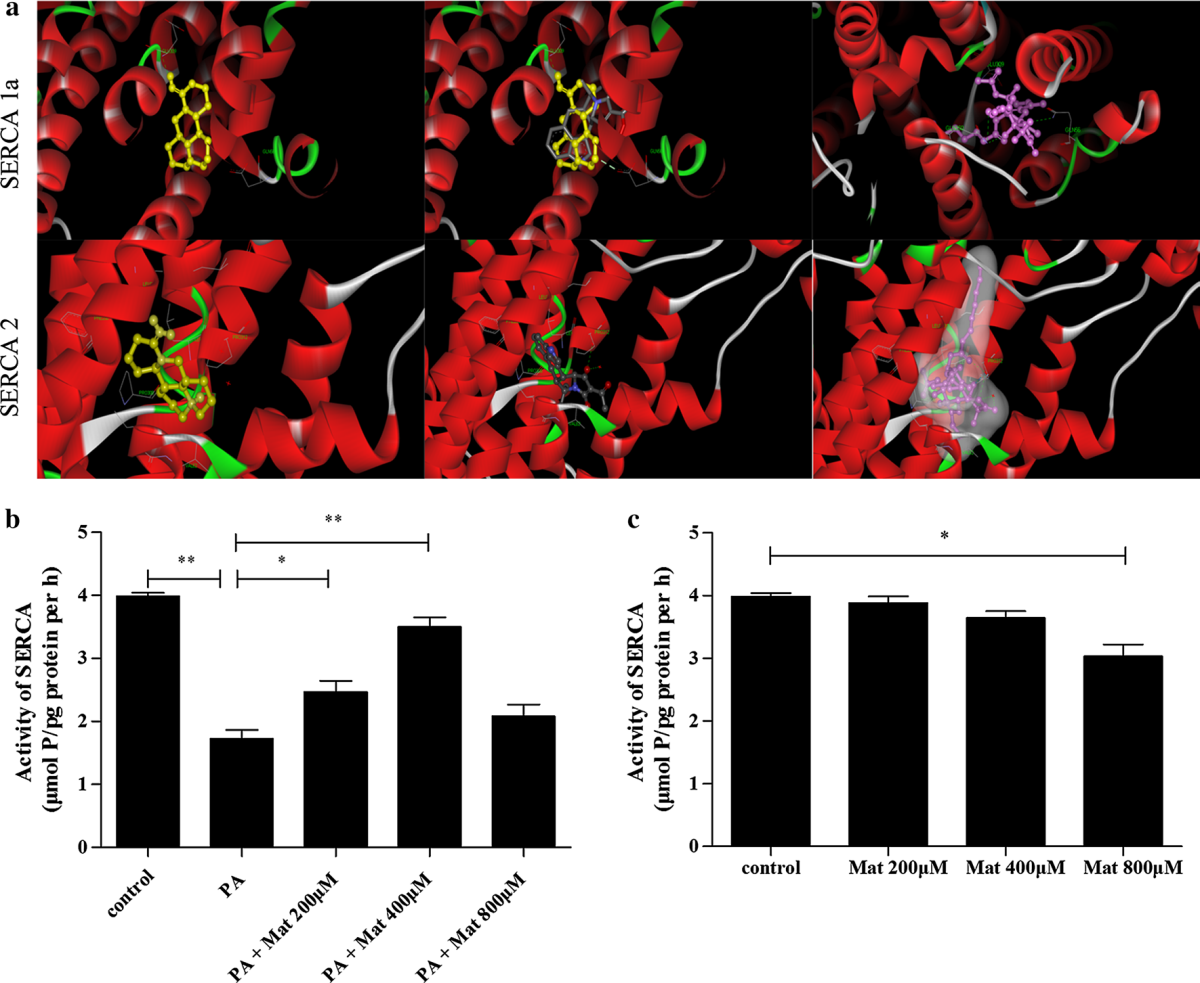
Mat modulates calcium homeostasis via the SERCA pathway. **a** computation docking of SERCA1a or SERCA2 with Mat (depicted in colored yellow). CPA (depicted in colored grey) and Tg (depicted in colored purple). In the activity test of SERCA (**b** and **c**), the L02 cells were treated with PA (500 μM), Mat (200, 400, 800 μM) or the combination of PA (500 μM) and Mat (200, 400, 800 μM) for 12 h. **b** the SERCA activity of the combination of PA and Mat. **c** the SERCA activity of Mat incubated alone. *P < 0.05 and **P < 0.01

In order to confirm the effect of Mat on SERCA, we conducted molecular docking experiment. As shown in the upper portion of Fig. 7a, Mat is similar with cyclopiazonic acid (CPA), the eutectic ligand of SERCA1a, because they have the approximate binding modes and action sites, and all of them can form a H bond with SERCA 1a. In the combined pocket, the carbonyl group of Mat can form the H bond with the amino group of the GLU309 on SERCA1a; Tg can form the H bond with the amino group of the GLU309, GLU250 and GLU56 on SERCA1a; while the pyrrole structure of CPA forms the H bond with ASP59. ASP59 plays a more important role in the binding pocket. This may be the reason for the activity of Mat slightly weaker than CPA and Tg. Non-polar interactions of Mat are similar to CPA with the hydrophobic residues lining the binding pocket, such as LEU253, LEU61and PRO312, as well as ILE307. CPA is also a ligand of SERCA2. As shown in the lower portion of Fig. 7a, Mat and CPA are combined with SERCA2 protein in a similar way. Although both of them do not form a H bond with the SERCA2 protein, CPA can form a H bond with the water molecules in the protein. Tg can also form H bonds with water molecules, and the hydrophobic structure of the fat chain occupies a pipe type hydrophobic cavity composed of ILE315, LEU249, LEU235 and PRO337, which makes the binding force stronger. The non H bonds of Mat include the hydrophobic amino acids PHE256, PRO312, LEU311, LEU253, LEU61, VAL62 and PRO308 of the binding pocket. Failure to form H bonds and fail to occupy hydrophobic cavities as well as carotenoids may be the reason for the weaker action of Mat.

Furthermore, the activity test of SERCA showed that the reduced SERCA activity induced by PA (500 μM) was ameliorated by low and middle dose Mat treatment (Fig. 7b). However, when Mat was incubated alone in L02 cells, there were no significantly differences on SERCA activity among vehicle, low dose Mat and middle dose Mat, while the activity was found to be inhibited by high dose Mat (Fig. 7c). Based on all these facts, we speculate that Mat is a competitive inhibitor of SERCA.

## Discussion

Nonalcoholic fatty liver disease is a clinical pathological syndrome characterized by diffuse hepatocytic bullous steatosis. Levels of serum TG are statistically significantly higher in patients with NAFLD [30]. We demonstrated that Mat could effectively reduce serum TC and TG content in HFD mice. Obesity is a major risk factor for many health complications, and NAFLD has increased in parallel with obesity in the United States [31]. In the present study, Mat remarkably reduced body weight gain and lipid generation related protein levels in HFD-induced obese mice, which may prevent the progression of NAFLD to a certain extent. Since Mat treatment reduced body weight, all the resulting phenotypes such as reduction in hyperlipidemia, lipogenesis, steatosis might be secondary and may not be a direct effect mediated by Mat treatment. Therefore, in order to further investigate the effect of Mat on liver lipid metabolism, we examined the effect of Mat on liver lipid metabolism-related proteins in later experiments. Our results showed that Mat down-regulated the proteins levels of sterol regulatory element binding protein 1c (SREBP-1c), fatty acid synthase (FAS), and acetyl-coenzyme A-carboxylase (ACC) in HFD mice. NAFLD is a spectrum of pathologic changes in the liver that ranges from simple steatosis to NASH, early fibrosis, cirrhosis and can progress to hepatocellular carcinoma [32]. In most cases, ALT and AST are elevated in NASH [33]. Meanwhile, inflammatory response is an important feature of NASH and an important driving factor in its progression to liver fibrosis [21]. The elevated levels of inflammatory factors and aminotransferase were observed in MCD mice, while administration of Mat could attenuate this effect. Furthermore, the results of H&E and oil red O staining showed that Mat administration markedly improved liver injury and lipid accumulation in HFD and MCD mice. In this study, Mat exerted a certain protective effect against the progression from steatosis to steatohepatitis in HFD and MCD diet-fed mice.

ER is notable for its central roles in Ca^2+^ storage, lipid biosynthesis, protein sorting and processing. The ER stress signal is mainly mediated by the following three ER-transmembrane proteins: PERK, IRE1α, and ATF6α [34]. When the ER homeostasis is unbalanced, GRP78 is separated from the transmembrane protein by binding with unfolded or misfolded proteins, and then PERK, IRE1α and ATF6α and their downstream signaling pathways are activated, resulting in increased lipid synthesis and apoptosis [35]. Our work showed that ER stress was significantly inhibited by Mat in the livers of MCD diet-fed mice, as well as in PA-induced L02 cells. Moreover, the expression of p-IRE1, ATF6, GRP78, and p-PERK were up-regulated in the livers MCD diet-fed mice which were reduced by long-term administration of Mat. In addition, low dose and middle dose Mat treatment could inhibit the ER stress in PA-induced L02 cells as well. These results indicated that Mat could effectively attenuate excessive ER stress in NAFLD.

ER stress is the central link in the occurrence of NAFLD. Excessive ER stress, mitochondrial dysfunction and oxidative stress can form a vicious cycle, which accelerates the progression of NAFLD. Since PA induces mitochondrial depolarization and suppresses autophagic activity, the proportion of dysfunctional mitochondria may be increased [36]. Our results showed that low and middle dose Mat reduced the damage of mitochondria induced by PA in L02 cells. Both ER stress and mitochondrial damage can generate a large amount of ROS [37, 38]. In turn, excessive ROS aggravates ER stress and mitochondrial dysfunction [27, 39, 40]. Massive generation of mitochondrial ROS opens the mitochondrial permeability transition pore (PTP), promotes apoptosis and necrosis, and leads to hepatic fibrosis [10]. Severe or persistent ER stress can lead to cell apoptosis [28, 41]. We found that low and middle dose Mat reduced ROS formation and apoptosis induced by PA in L02 cells. These results indicated that low and middle dose Mat which could effectively attenuate mitochondrial damage, oxidative stress and apoptosis induced by excessive ER stress in NAFLD, but not high dose Mat.

Ca^2+^ is considered to play a key role in ER stress, mitochondrial dysfunction, oxidative stress and liver insulin resistance, all of which are important features of NAFLD [7, 25, 42, 43]. The destruction of cytosolic Ca^2+^ balance, especially the excessive release of ER Ca^2+^, is the core factor to lead to ER stress, mitochondrial damage and a series of problems that followed. The SERCA pump is a key site to regulate the ER calcium balance, because it participates in the redistribution of Ca^2+^ from cytoplasm to ER. Recent studies showed that PA induced ER stress, apoptosis and accompanied by a reduction in SERCA2b activity, mimicking nonalcoholic steatohepatitis [7, 44]. We observed that cytosolic Ca^2+^ levels were increased in PA-induced L02 cells after 12 h incubation, while Mat treatment attenuated this effect. This result indicated that Mat was involved in Ca^2+^ regulation in NAFLD. In order to further clarify the role of Mat, we conducted a series of instantaneous stimulation experiments in normal L02 cells. Firstly, we found that Mat stimulation increased [Ca^2+^]_c_ in a concentration-dependent manner and this effect was partially suppressed when combined with 2-APB (IP_3_R inhibitor). However, the effect was mostly suppressed in cells after Tg (SERCA inhibitor) treated 12 h. Studies have pointed out that 2-APB reduces ER Ca^2+^ when ER Ca^2+^ store is replete [29]. We also observed that there was a slight increase when 2-APB (20 μM) stimulated L02 cells and the phenomenon disappeared when cells were stimulated with Mat in advance for 10 min. This indicated that Mat treatment reduced the Ca^2+^ concentration in ER, which is a similar effect with Tg. Moreover, we found that Tg stimulation was able to increase the cytosolic Ca^2+^ level while Mat and Tg simultaneous stimulation showed a lower Ca^2+^ wave than that in Tg stimulation. As is known to all, the calcium exchange between the ER and the cytoplasm depends mainly on three calcium channels, including IP_3_R, RyR and SERCA. RyR has a poor effect on liver cells for it is mainly located in cardiac muscle and skeletal muscle. Our stimulation tests were carried out in a non-calcium environment, which excludes the influence of external calcium ions. Therefore, we suspected that Mat was likely involved in calcium regulation in the SERCA pathway. In order to verify this, we conducted molecular docking experiment. Among the entire cluster of complexes for SERCA1a and SERCA2 predicted by AutoDock, the most populated cluster conformations together with the lowest energy conformation show that Mat might also function as a SERCA pump inhibitor. Furthermore, SERCA activity assay showed that Mat up-regulated SERCA activity in PA-induced L02 cells, although this enzyme activity might be reduced when high dose Mat was used in control group. These results supported that Mat mitigated the ER stress of NAFLD through competitive inhibition of SERCA.

In the present study, the highest dose of Mat in mouse models was converted from clinical common dose in people and all doses of Mat showed a beneficial effect. However, in PA-induced L02 cells, high dose Mat has different pharmacological effects from middle and low dose Mat, which seems to be contradictory. In fact, the diverse pharmacological effects of Mat on ER stress have been reported in the previous literature. On the one hand, hepatitis B and atherosclerosis are considered to be associated with excessive ER stress, and Mat has a good therapeutic effect on these two diseases [45–48]. On the other hand, some researchers have discovered that Mat also exerts anti-cancer effects by aggravating ER stress [49]. The increase of SERCA2 activity was demonstrated in cancer cells and is considered to protect cancer cells from apoptosis [50]. Our experiments show that the anti-cancer effects of Mat may be exerted by inhibiting SERCA2. In some other diseases, in spite of the stress, Mat as a inhibitor of SERCA, it can exert its competitive inhibitory effect to improve SERCA activity and maintains the ER function, thereby improving the state of the disease.

## Conclusion

Data from this study support the view that Mat, a herbal medicine-derived compound, clearly attenuated liver steatosis and steatohepatitis in HFD and MCD mice in vivo and in cultured L02 cells in vitro when it was used at a suitable concentration. By functioning as a competitive inhibitor of SERCA, Mat is able to improve the state of ER stress, and then alleviates lipid metabolic disorder, mitochondrial dysfunction and inflammatory reaction, all of which participate in NAFLD pathological progression. Our findings make a possible explanation for the contradictory effects of Mat on ER stress in the previous literature and suggest the use of Mat as a potential therapeutic strategy for NAFLD.

## Additional file

**Additional file 1: Figure S1.** The body weight of HFD mice at 0th, 4th, 8th, and 12th. **Figure S2**. The cellular contents of TG and TC in cellular model.

## Abbreviations

2-APB: 2-aminoethoxydiphenyl borate
ACC: acetyl-coenzyme A-carboxylase
ATF6: activating transcription factor 6
CHOP: CCAAT/enhancer-binding protein homologues protein
CPA: cyclopiazonic acid
FAS: fatty acid synthase
GRP78: glucose-regulated protein 78
HFD: high-fat-diet
IL-6: interleukin-6
IL-10: interleukin-10
IRE1: inositol-requiring enzyme 1
JNK: c-Jun N-terminal kinase
Mat: matrine
MCD: methionine–choline-deficient
NAFLD: nonalcoholic fatty liver disease
NASH: nonalcoholic steatohepatitis
NF-κB: nuclear factor-k-gene binding
PA: palmitic acid
PERK: the PKR-like ER protein kinase
RIPA: radioimmune protection assay
SERCA: sarcoplasmic/endoplasmic reticulum calcium ATPase
SREBP1c: sterol regulatory element binding protein 1c
TC: total cholesterol
TG: triglyceride
Tg: thapsigargin
TNF-α: tumor necrosis factor alpha
