# Correction to: Matrine attenuates endoplasmic reticulum stress and mitochondrion dysfunction in nonalcoholic fatty liver disease by regulating SERCA pathway

**DOI:** 10.1186/s12967-019-2020-2

**Published:** 2019-08-21

**Authors:** Xiaobo Gao, Shun Guo, Song Zhang, An Liu, Lei Shi, Yan Zhang

**Affiliations:** Department of Pharmacy, The Second Affiliated Hospital of Air Force Medical University, 569 N. Xinsi Road, Xi’an, 710038 China

## Correction to: J Transl Med (2018) 16:319 10.1186/s12967-018-1685-2

Following publication of the original article [1], the authors reported errors in Fig. 5. The ROS picture of low dose Marine intervention group in Fig. 5d was used incorrectly, which was caused by the error of the storage path of the picture in the experiment. It was not discovered in time due to the approximation of the two graphs. In addition, the label of middle dose Marine intervention group in Fig. 5a was omitted.

In this Correction the incorrect and corrected version of Fig. 5 are shown.

Originally Fig. 5 was published as:

**Fig. 5:**
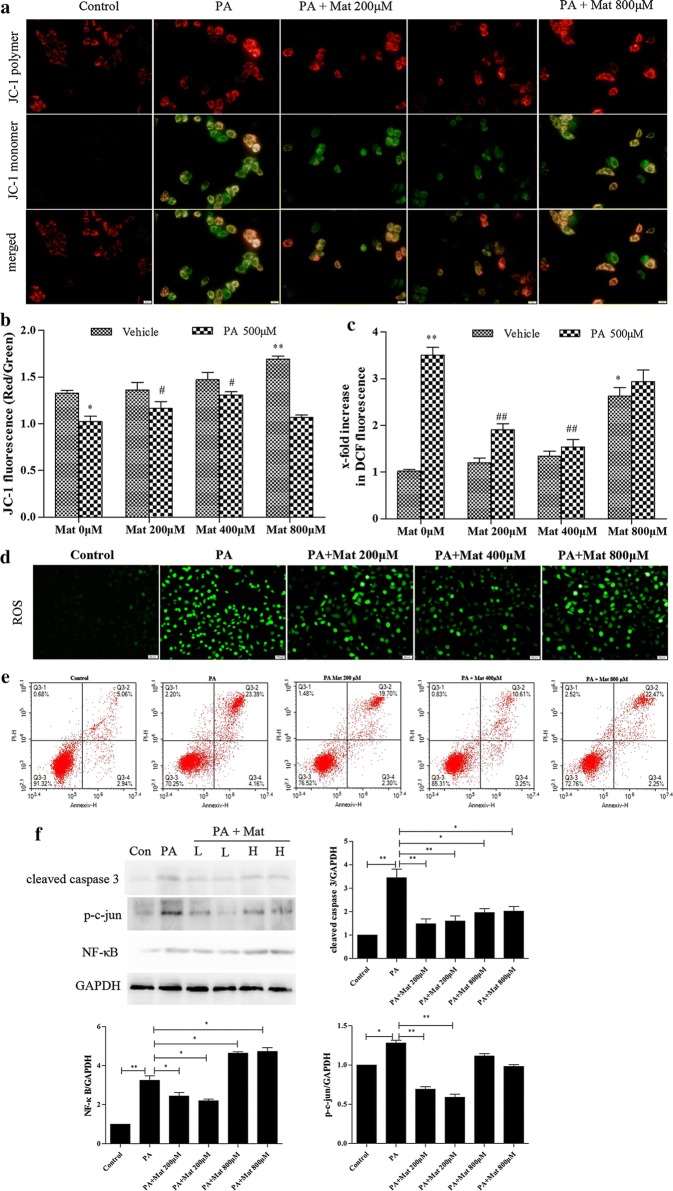
Effect of Mat on mitochondrial activation, ROS production and apoptosis in PA-induced L02 cells. The L02 cells were treated with PA (500 μM), Mat (200, 400, 800 μM) or the combination of PA (500 μM) and Mat (200, 400, 800 μM) for 12 h. **a** Mitochondrial membrane potential (MMP) imaging (×400). **b** JC-1 fluorescence and **c** DCF fluorescence detected by fluorescence spectrophotometer. *P < 0.05 and **P < 0.01 vs. Control, ^#^P < 0.05 and ^##^P < 0.01 vs. PA. **d** ROS imaging (×400). **e** apoptosis analyzed by flow cytometry. **f** Expression of cleaved caspase 3, p–c-jun and NF-κB in L02 cells. *P < 0.05 and **P < 0.01

The corrected version of Fig. 5:

**Fig. 5:**
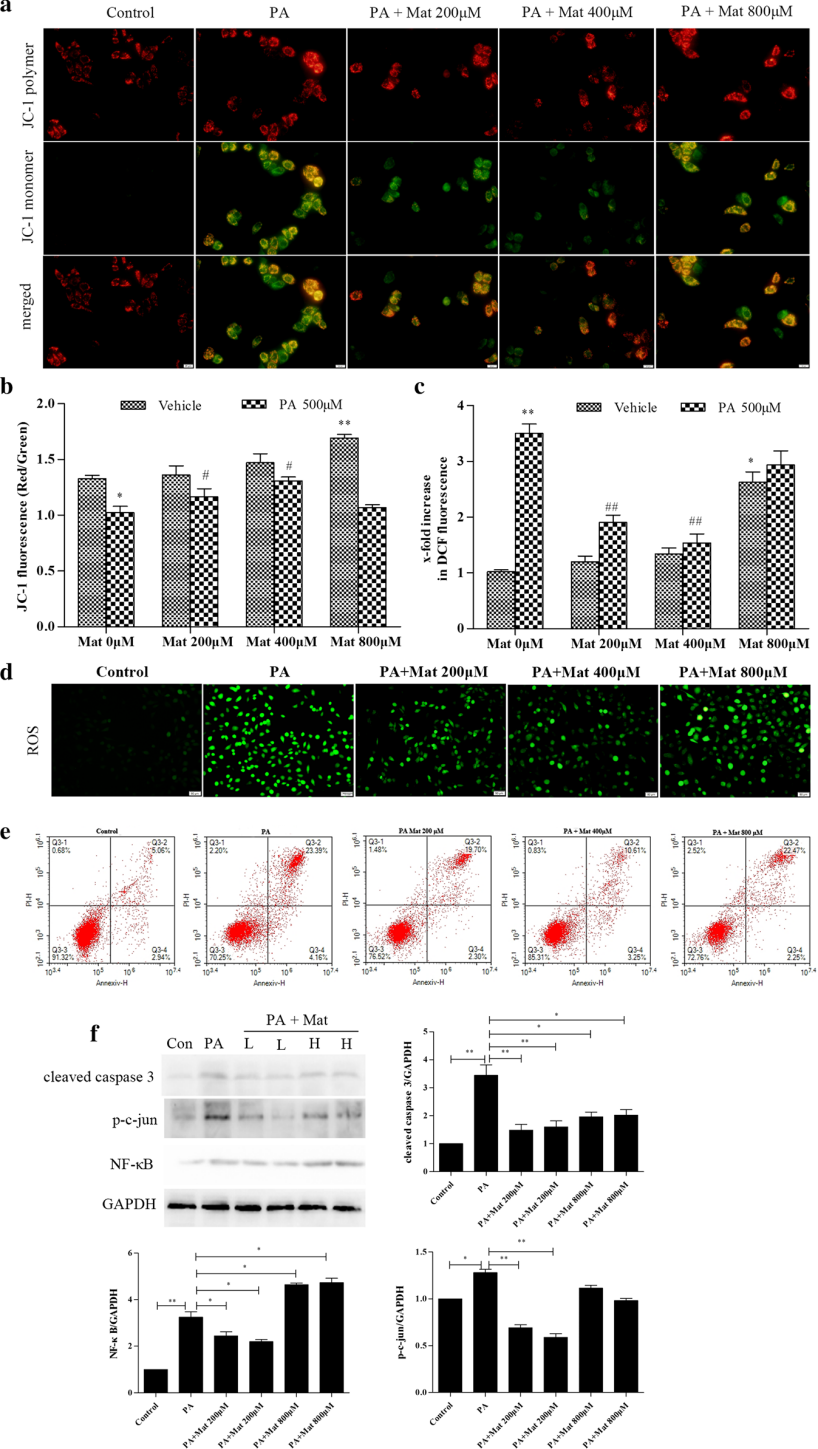
Effect of Mat on mitochondrial activation, ROS production and apoptosis in PA-induced L02 cells. The L02 cells were treated with PA (500 μM), Mat (200, 400, 800 μM) or the combination of PA (500 μM) and Mat (200, 400, 800 μM) for 12 h. **a** Mitochondrial membrane potential (MMP) imaging (×400). **b** JC-1 fluorescence and **c** DCF fluorescence detected by fluorescence spectrophotometer. *P < 0.05 and **P < 0.01 vs. Control, ^#^P < 0.05 and ^##^P < 0.01 vs. PA. **d** ROS imaging (×400). **e** apoptosis analyzed by flow cytometry. **f** Expression of cleaved caspase 3, p–c-jun and NF-κB in L02 cells. *P < 0.05 and **P < 0.01
