# Supplementary material for: Matrine attenuates endoplasmic reticulum stress and mitochondrion dysfunction in nonalcoholic fatty liver disease by regulating SERCA pathway
Source: J Transl Med. 2018 Nov 20;16:319. doi: 10.1186/s12967-018-1685-2 (PMC6245862; doi:10.1186/s12967-018-1685-2)
Supplement: Supplementary file 1 — Additional file 1: Figure S1. The body weight of HFD mice at 0th, 4th, 8th, and 12th. Figure S2. The cellular contents of TG and TC in cellular model. [file 12967_2018_1685_MOESM1_ESM.doc]

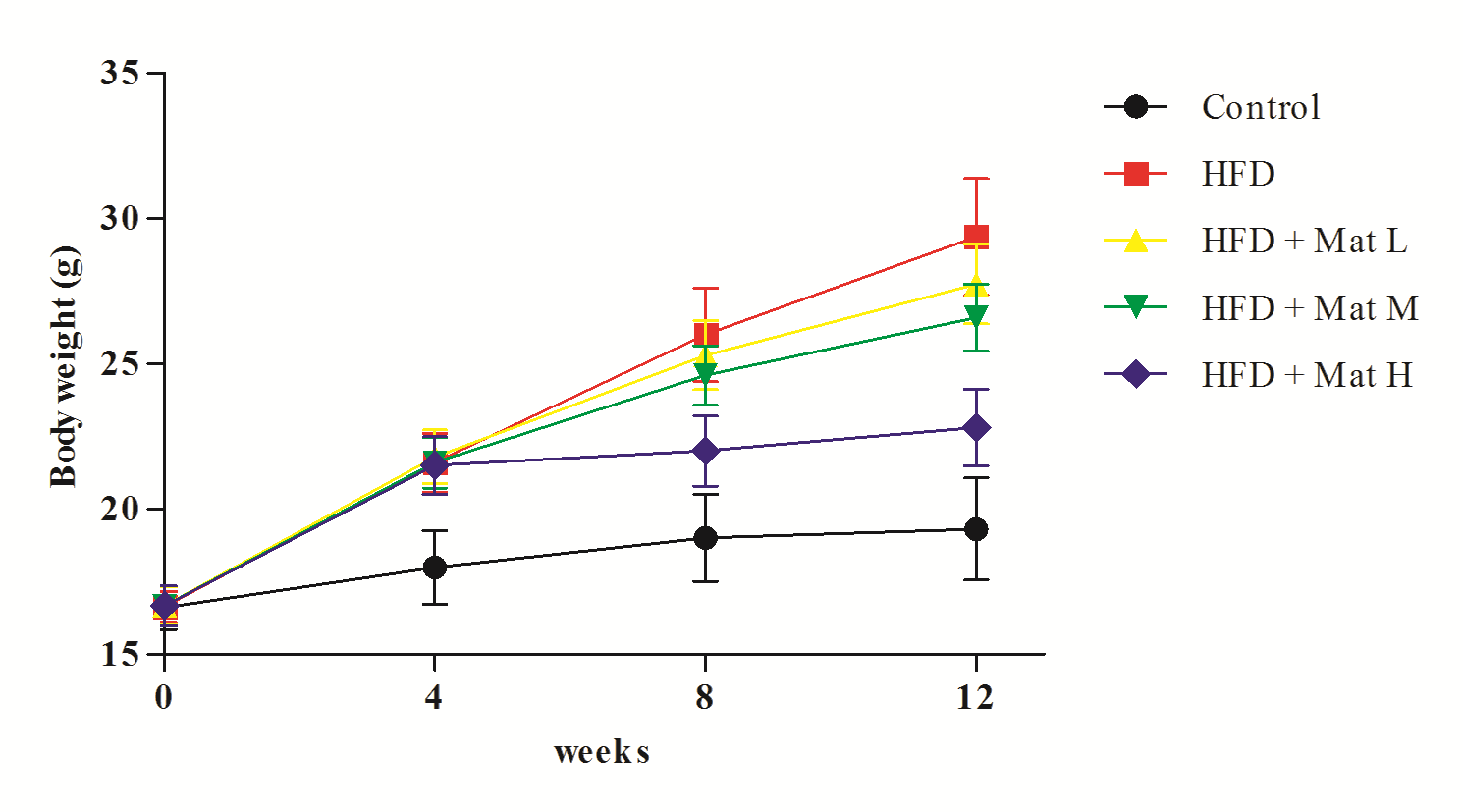


Figure S1. The body weight of HFD mice at 0th, 4th, 8th, and 12th.


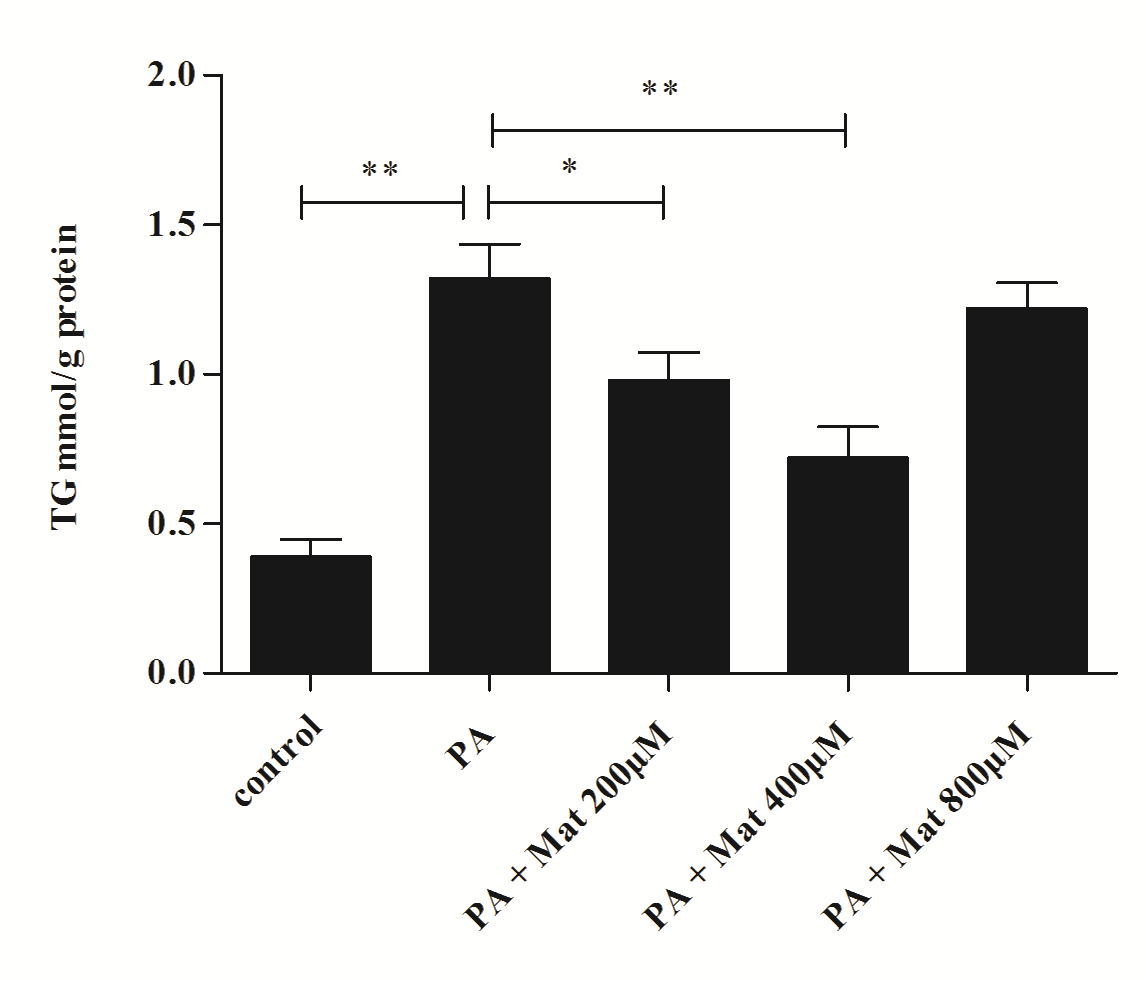

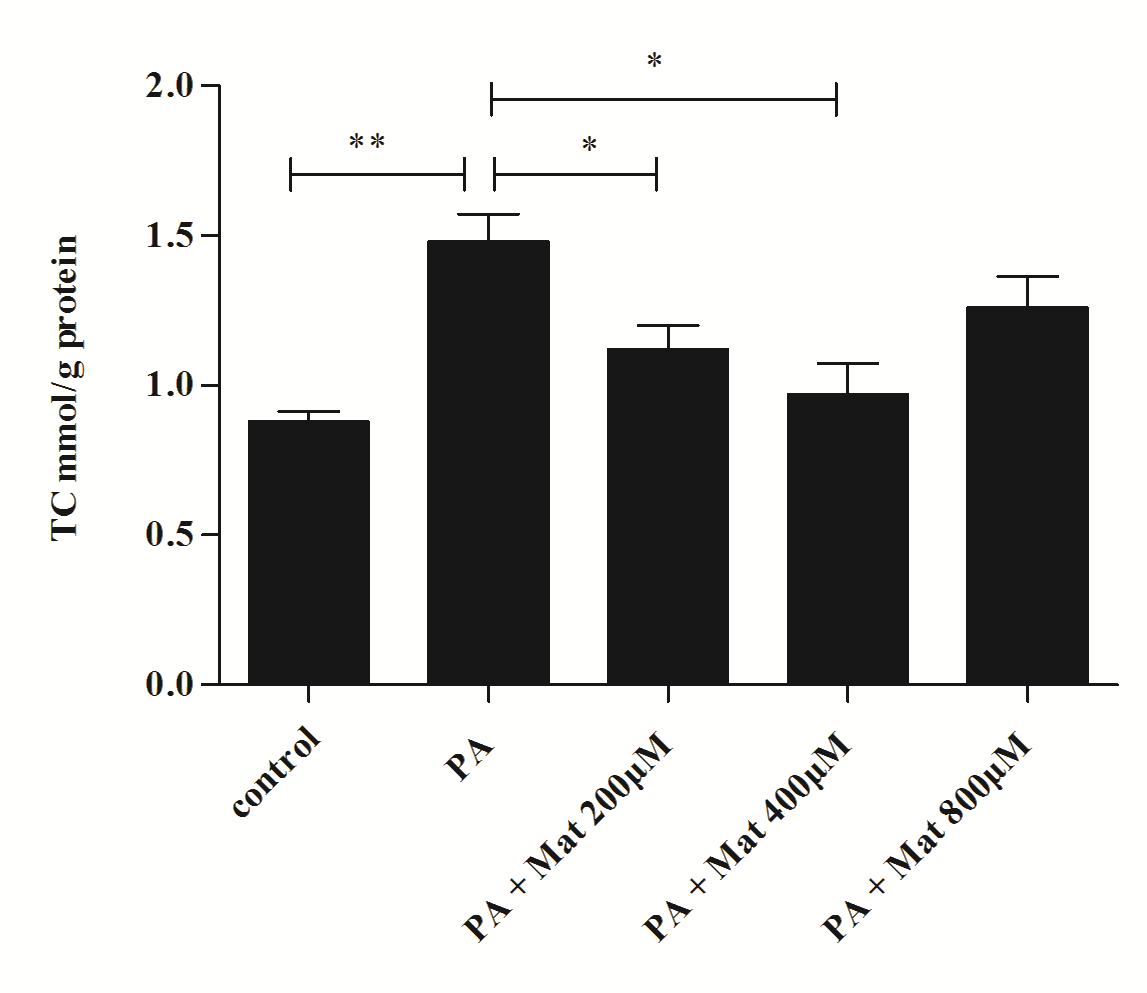


Figure S2. The L02 cells were treated with PA (500 μM) or the combination of PA (500 μM) and Mat (200,400,800 μM) for 12 h. then cellular contents of TG and TC were determined by commercial kits.
